# Application of self-organised learning environments integrated with generative AI in standardised training for residents

**DOI:** 10.3389/fmed.2026.1752647

**Published:** 2026-03-16

**Authors:** Haiping Luo, Hongmin Yu

**Affiliations:** 1 Department of Gastrointestinal and Anorectal Surgery, Huangshi Central Hospital, Huangshi, Hubei, China; 2 Department of Breast and Thyroid Surgery, Ward 2 (Clinical Skills Training Center), Huangshi Central Hospital, Huangshi, Hubei, China

**Keywords:** digital literacy, generative AI (GenAI), self-organised learning environments, teaching effectiveness, teaching satisfaction

## Abstract

**Objective:**

This study aimed to evaluate the effectiveness of the GAiSOLEs teaching model, which integrates Self-Organised Learning Environments (SOLEs) with Generative Artificial Intelligence (GAI), in improving teaching effectiveness, digital literacy and teaching satisfaction in the standardized training of residents.

**Methods:**

A single-center, double-blind randomized controlled trial was conducted in this study. A total of 114 standardized training participants for rotating residents were enrolled and randomly divided into two groups: the intervention group (GAiSOLEs teaching model, *n* = 57) and the control group (traditional teaching model, *n* = 57). The primary outcome was the teaching effectiveness score before and after intervention. Secondary outcomes included digital literacy score and teaching satisfaction score, both evaluated by standardized scales. Data analysis was performed using *χ*^2^ test. Paired and independent samples *t*-tests, Mann–Whitney U test, and hierarchical multiple linear regression.

**Results:**

Post-intervention teaching effectiveness scores were significantly elevated relative to baseline levels in both the intervention and control groups (all *p* < 0.05). For all dimensions of teaching effectiveness, the gain scores in the intervention group were markedly higher than those in the control group, with large effect sizes observed across all metrics (theoretical knowledge test: *p* < 0.001, Hedges’ g = 1.0; clinical skill operation: *p* < 0.001, Hedges’ g = 1.87; standardized medical record writing: *p* < 0.001, Hedges g = 1.16; clinical thinking ability: *p* < 0.001, Hedges’ g = 1.21; doctor-patient communication skills: *p* < 0.001, Hedges’ g = 1.27). Hierarchical linear regression analysis, after adjusting for covariates including gender, educational background, professional distribution and pre-test scores, revealed that the GAiSOLE intervention (versus the control condition) was a significant positive predictor of higher scores for all five training outcomes (all *p* < 0.05): theoretical examination (*β* = 2.329, ΔR^2^ = 0.294), clinical skill operation (*β* = 2.947, ΔR^2^ = 0.499), standardized medical record writing (*β* = 3.366, ΔR^2^ = 0.331), clinical thinking ability (*β* = 3.391, ΔR^2^ = 0.412), and doctor-patient communication competence (*β* = 3.157, ΔR^2^ = 0.355). Moreover, the experimental group achieved significantly higher scores than the control group across all dimensions of digital literacy (all *p* < 0.05). Additionally, the group also reported significantly higher satisfaction ratings for all teaching parameters relative to the control group (all *p* < 0.05).

**Conclusion:**

The GAiSOLE teaching model was associated with improved teaching effectiveness, digital literacy and teaching satisfaction among residents, and thus holds promising potential as a novel approach for the standardized training of residents.

## 1 Background

Standardized Residency Training (SRT) is the core of China’s medical education, bridging medical theory and clinical practice. The professional literacy and clinical skills of physicians trained through this program are directly linked to the quality of medical services and patients’ health rights (1). However, the current system has three prominent deficiencies, making it difficult to meet modern clinical needs: first, a rigid model—"teacher-led instruction with trainee passive reception”—lacks hierarchical design tailored to individual differences; second, overemphasis on one-way knowledge indoctrination neglects trainees’ subjectivity, weakening their active learning and innovative capabilities; third, inadequate cultivation of digital literacy, with no systematic curricula or training integrated with clinical digital scenarios, resulting in trainees’ insufficient ability to adapt to intelligent diagnostic and therapeutic tools (2, 3). It is difficult to meet the dynamically evolving demands of the digital intelligence era (4).

Rooted in constructivist theory, SOLEs adopt a learner-centered approach, constructing flexible scenarios to facilitate independent and collaborative inquiry (5). Validated across global educational contexts spanning primary to higher education—including STEM education in South Africa, EFL instruction in Oman, and programming logic courses in Indonesia—SOLEs effectively stimulate learning initiative and creativity, which aligns with the competency-oriented concept of modern medical education (5–8). GAI complements SOLEs through precise feedback mechanisms such as real-time personalized suggestions for clinical operation errors and efficient resource integration capabilities including the delivery of tailored clinical cases and updated practice guidelines. This synergy addresses the key shortcomings of traditional teaching, namely delayed feedback and insufficient personalization, with findings consistent with international research on technology-integrated SOLE applications (9–11).

Against the backdrop of clinical digital transformation and identified deficiencies in traditional SRT, this study intends to develop and evaluate the GAiSOLEs model, an integration of constructivist theory and the technical merits of GAI. With clinical residents’ SRT as a case study, the model will examine its effects on trainees’ clinical knowledge acquisition, digital literacy, and training satisfaction. A randomized controlled trial will be implemented to compare the efficacy of this novel model with that of the traditional SRT model, with a focus on the application effectiveness of integrating GAI and SOLEs in residency training. Ultimately, the model is anticipated to offer innovative perspectives and a practical basis for the digital transformation and quality enhancement of SRT.

## 2 Objects and methods

### 2.1 Research objects

**Figure 1:**
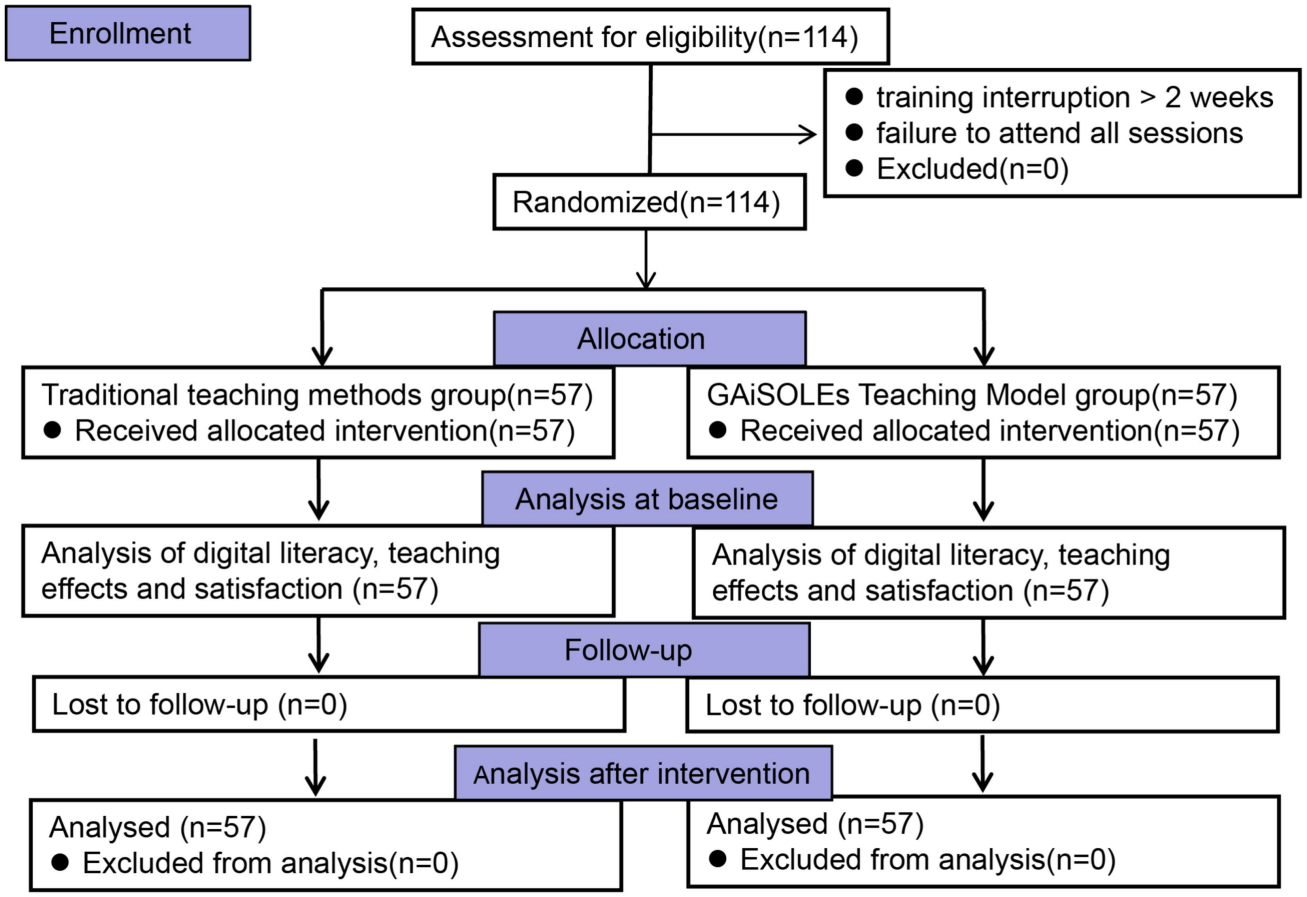
Flowchart of the study.

A total of 114 residents receiving standardised training at Huangshi Central Hospital from March 2025 to July 2025 were enrolled in this study. Participants were randomly assigned in a 1:1 ratio to the intervention group (GAiSOLEs teaching model, *n* = 57) or the control group (traditional teaching model, *n* = 57). Inclusion criteria: ① Meeting the admission conditions for standardised residency training and formally entering the training system; ② Voluntarily participating in this study and signing the informed consent form; ③ Completing the full training cycle without dropping out or taking leave midway; ④Having basic computer operation and network retrieval abilities. Exclusion criteria: ①Had participated in trials related to the GAiSOLE teaching model; ② Those who interrupted training due to illness, job transfer and other reasons; ③Suffered from severe physical or mental illnesses; ④Failed to sign the informed consent form. All participants volunteered and could withdraw at any time. The flowchart of the study is shown below (Figure 1). This study was conducted after being approved by the Medical Ethics Committee of Huangshi Central Hospital (Project No.: 2024011801).

### 2.2 Allocation, randomization and anti-contamination measures

A full-time professional from the clinical skills training center generated a central random allocation sequence using an online random-number generator^1^ before participant enrollment. Allocation concealment was achieved using sequentially numbered, sealed, opaque envelopes. Envelopes were opened only after participants completed eligibility screening, signed informed consent, and finished baseline assessments, ensuring recruiters and assessors were blinded to group assignments. To minimize contamination bias, three measures were adopted: (1) An independent online learning platform with exclusive access was established for the intervention group, with no control group access; (2) Anti-contamination protocol clauses, confidential informed consent, and teacher-specific standardized operating procedures (no intervention-specific content for the control group) were implemented; (3) Outcome assessors were blinded to group allocation to reduce assessment bias.

### 2.3 Construction of GAiSOLEs teaching model

#### 2.3.1 GAiSOLEs platform construction

The SOLEs platform, a fully independent online learning platform built on the hospital’s existing online education system, is an exclusive self-organized learning platform that integrates four core modules: resource library, collaboration space, discussion area, and achievement display area. Each module is designed around the learning needs of trainees, with clear functions and distinct divisions of labor.

Among them, the resource library integrates clinical cases, diagnosis and treatment guidelines, literature materials, and operation videos to provide trainees with comprehensive and standardized learning materials; the collaboration space supports trainees to form groups independently and carry out online and offline collaborative inquiry to improve their mutual learning and problem-solving abilities; the discussion area provides a communication channel for trainees, allowing them to deepen their understanding of professional knowledge through questioning, consulting, sharing experiences, and ideological exchanges; the achievement display area encourages trainees to share learning experiences, case reports and other achievements to promote mutual progress.

To ensure the intervention effect and achieve strict group isolation, all specific tools and resources of this platform can only be accessed through dedicated tablet computers uniformly managed by the Clinical Skills Training Center. Moreover, the activation licenses for the tablets are only issued to members of the intervention group, enabling them to access modules containing novel teaching strategies and artificial intelligence tools. In contrast, the control group has no access to this platform and its supporting resources, effectively ensuring the isolation of intervention measures.

#### 2.3.2 Introduction of GAI tools

The DeepSeek-V3-0324 model was selected, and this AI tool mainly involves learning plan formulation, medical knowledge Q&A, case analysis guidance and simulated diagnosis and treatment training. As virtual cases were utilized in this study, no identifiable private or sensitive patient data were involved. Accordingly, de-identification procedures, data usage restrictions, and relevant AI governance and data protection protocols were not applicable in the present study.

#### 2.3.3 Teacher training

Including SOLEs teaching philosophy, AI usage methods and learning process guidance skills.

#### 2.3.4 Trainee training

Participants in the experimental group received training on AI tool operation and guidance on self-organised learning, which equipped them with platform operation skills, group collaboration norms, and independent inquiry abilities. The generative AI tool was applied solely for educational training to assist case analysis and enhance clinical thinking, not for clinical diagnosis or treatment. All participants were informed that only virtual cases were used, with no real patient data or privacy risks. The informed consent form documented the AI tool’s information, limitations, and that its outputs were for teaching reference only.

### 2.4 Research processes

Participants in the intervention and control groups completed their learning in parallel, with both groups following the same learning schedule: 90 min per session, two sessions per week, and a total of 30 sessions.

#### 2.4.1 Specific implementation steps of GAiSOLEs teaching model

the GAiSOLEs Teaching Model consists of six sequential steps: Initiation of Self-Organized Learning, AI-Assisted Case Generation & Independent Inquiry, Result Verification and Hallucination Avoidance, AI-Assisted Expansion of Difficult Cases, Self-Organized Summary and Reflection, and Evaluation and Feedback. Taking acute appendicitis as an example, the six sequential steps are elaborated in detail (see Table 1; Supplementary material S3).

**Table 1:** Specific implementation steps of GAiSOLEs teaching model.

Implementation step	Core operations
Step 1: Initiation of self-organized learning	1. Teacher clarifies teaching goals and AI application scope 2. Residents form 4-person groups, confirm roles and formulate learning schedules 3. AI-assisted preview (generate outline and mark key questions)
Step 2: AI-Assisted case generation and independent inquiry	1. AI generates an advanced atypical acute appendicitis case 2. Group sorts out case information, discusses diagnosis and obtains AI guidance 3. Formulates preliminary diagnosis and treatment strategy
Step 3: Result verification and hallucination avoidance	1. Group verifies 5 core points, corrects errors and compiles a preliminary report 2. AI-assisted verification and hallucination avoidance; 3. Final verification to form a formal report, teacher conducts random spot checks
Step 4: AI-Assisted expansion of difficult cases	1. AI generates a periappendiceal abscess case 2. Group discusses complication management, obtains AI guidance and completes verification according to Step 3
Step 5: Self-organized summary and reflection	1. Group summarizes diagnosis and treatment process, core difficulties and AI application experience 2. AI-assisted review of high-frequency errors 3. Individual reflection and formulation of follow-up learning plans
Step 6: Evaluation and feedback	1. Group mutual evaluation and self-assessment; 2. AI generates 5 case analysis questions with reference answers 3. Teacher summarizes, guides AI use and completes the teaching cycle

#### 2.4.2 Control group

Traditional teaching methods for standardised residency training were adopted, mainly including: ①Theoretical teaching: Adopting offline classroom or special lecture forms. ②Clinical practice: Trainees follow supervising teachers to participate in clinical diagnosis and treatment work and practice clinical skill operations. ③Case discussion: Led by supervising teachers with trainees’ participation. ④Assessment and evaluation: Theoretical and operational skill assessments are conducted in accordance with the requirements of the standardised training syllabus to feedback learning effects.

### 2.5 Outcomes

Prior to study initiation, a pilot investigation was administered among 10 residents enrolled in the standardized residency training program to evaluate the reliability of all questionnaires and scales employed in this study. Following the completion of the intervention, outcome assessors were fully blinded to group allocation and remained unaware of group assignment throughout the assessment and scoring procedure, thereby minimizing observer bias and detection bias. In the present study, inter-observer reliability was excellent, with an intraclass correlation coefficient (ICC) of 0.85 for continuous clinical variables and a Cohen’s kappa coefficient of 0.80 for categorical outcomes. All scoring discrepancies were resolved via consensus discussion among the assessors; any unresolved disagreements were adjudicated by an independent senior clinical reviewer. Detailed evaluation rubrics, standardized scoring criteria, and assessment protocols are presented in Supplementary materials S1, S2.

#### 2.5.1 Primary outcomes

##### 2.5.1.1 Teaching effect evaluation

①Theoretical knowledge test: Test questions were designed in accordance with the standardised training syllabus, with a closed-book exam and a full score of 100 points; ② Clinical skill assessment: An assessment team consisting of 3 teachers with residency training teacher qualifications was formed, and the assessment content included medical record writing and skill operation, each with a full score of 100 points (Supplementary material S1); ③ Clinical thinking ability score: A scoring table was formulated with reference to literature, consisting of case analysis questions in the theoretical exam (accounting for 60%) and “diagnostic logic” in medical record writing (accounting for 40%, Supplementary material S2); ④ Doctor-patient communication ability score: The SEGUE Framework for Assessing Doctor-Patient Communication Skills was used for evaluation, which includes 5 dimensions and 25 items: communication initiation, information collection, information giving, understanding patients and closing the consultation. During the communication process, 1 point was awarded for each occurrence of communication content items, and 0 points for each failure to perform communication skill items (12). The SEGUE scale demonstrated excellent reliability in this study, with an overall Cronbach’s *α* of 0.891, split-half reliability of 0.803, and test–retest ICC of 0.833. Gain score was calculated as post-intervention score minus pre-intervention score.

#### 2.5.2 Secondary outcomes

##### 2.5.2.1 Digital literacy evaluation

The digital literacy of physicians was assessed using the Standardised Residency Training Trainee Digital Literacy Evaluation Scale, which covers 5 dimensions including digital application, digital social responsibility, digital awareness, professional development and digital technology knowledge and skills, with a total of 33 items (13). Each item was scored using a 5-point Likert scale (1 = strongly disagree, 5 = strongly agree), and higher scores for each dimension and total score indicate a higher level of digital literacy. The Digital Literacy Evaluation had a Cronbach’s *α* of 0.870, split-half coefficient of 0.820 and test–retest ICC of 0.813, showing good reliability and internal consistency in this study.

##### 2.5.2.2 Teaching satisfaction questionnaire evaluation

A modified teaching satisfaction questionnaire referenced from literature was used, covering 3 modules: self-capability improvement (3 items), teaching model evaluation (4 items) and teacher satisfaction (2 items). Each module was scored on a scale of 0–10 points (12). The Cronbach’s *α* coefficient of the teaching satisfaction questionnaire was 0.861, the split-half reliability coefficient was 0.848, and the test–retest ICC was 0.825, indicating high internal consistency and satisfactory reliability in the present study.

### 2.6 Statistical methods

All statistical analyses in this study were performed using SPSS 24.0 software (IBM Corp., Armonk, NY, USA). Continuous variables were presented as mean ± standard deviation (SD), and categorical variables as *n* (%). The Shapiro–Wilk test was used to assess the normality of data distribution. For continuous data, the normality test was conducted first: the independent-samples *t*-test was applied for intergroup comparisons if the data conformed to a normal distribution with homogeneous variances; otherwise, the nonparametric Mann–Whitney U test was used. For categorical data, the chi-square test was adopted for unordered data. Adjusted means and effect sizes (Hedges’s g) were reported, with the magnitude of effect classified by the absolute value of g: |g| < 0.2 indicating a small effect, 0.2 to 0.8 a moderate effect, and ≥0.8 a large effect. Hierarchical multiple linear regression analysis was used to examine the effect of the GAiSOLEs Teaching Model on residents’ Teaching Effect scores (primary outcome variable), with adjustments made for confounding factors. The goodness of model fit was evaluated using the coefficient of determination (*R*^2^), change in *R*^2^ (Δ*R*^2^), and F statistic. The primary reported indicators included the standardized regression coefficient (*β*), t value, and *p* value. All tests were two-tailed, and a *p* value < 0.05 was considered statistically significant. *p* values were adjusted for multiple comparisons using the Holm method.

## 3 Results

### 3.1 Comparison of general data of residents in the two groups

There were 57 trainees in each group. In the control group, there were 26 males (45.61%) and 31 females (54.39%); the average age was (25.36 ± 1.72) years; educational background composition: 38 undergraduates (66.67%), 19 postgraduates (33.33%); professional distribution: 20 in internal medicine (35.08%), 16 in surgery (28.07%), 8 in obstetrics and gynaecology (14.04%), 5 in pediatrics (8.77%), and 8 in other departments (14.04%). In the experimental group, there were 24 males (42.11%) and 33 females (57.89%); the average age was (25.14 ± 1.85) years; educational background composition: 37 undergraduates (64.91%), 20 postgraduates (35.09%); professional distribution: 19 in internal medicine (33.33%), 17 in surgery (29.82%), 8 in obstetrics and gynaecology (14.03%), 6 in pediatrics (10.52%), and 7 in other departments (12.30%). There were no statistically significant differences in gender (*χ*^2^ = 0.143, *p* = 0.706), age (*t* = 0.665, *p* = 0.507), educational background (*χ*^2^ = 0.043, *p* = 0.836) and professional distribution (*χ*^2^ = 0.176, *p* = 0.995) between the two groups, with comparability.

### 3.2 Comparison of teaching effect scores of trainees in the two groups

Before the teaching intervention, no statistically significant differences were observed between the two groups in all assessed dimensions (Table 2). Specifically, the experimental and control groups showed the following scores and statistical results: theoretical knowledge test (83.33 ± 1.12 vs. 82.65 ± 1.18, *t* = 0.497, *p* = 0.620), clinical skill operation (81.84 ± 1.32 vs. 82.05 ± 1.01, *t* = 0.957, *p* = 0.341), medical record writing standardisation (83.25 ± 2.28 vs. 83.40 ± 1.96, *t* = 0.129, *p* = 0.897), clinical thinking ability (81.98 ± 2.12 vs. 81.65 ± 1.90, *t* = 0.942, *p* = 0.346), and doctor-patient communication skills (10.11 ± 1.45 vs. 10.21 ± 1.28, *t* = 0.595, *p* = 0.552).

**Table 2:** Comparison of pre-and post-test outcomes for experimental and control groups.

Dimensions	Experimental	Control	*t* value	*p* value
Theoretical knowledge test score	83.33 ± 1.12	82.65 ± 1.18	0.497	0.620
Clinical skill operation	81.84 ± 1.32	82.05 ± 1.01	0.957	0.341
Medical record writing standardisation	83.25 ± 2.28	83.40 ± 1.96	0.129	0.897
Clinical thinking ability	81.98 ± 2.12	81.65 ± 1.90	0.942	0.346
Doctor-patient communication skills	10.11 ± 1.45	10.21 ± 1.28	0.595	0.552

After the intervention, the experimental group had significantly higher gain scores than the control group across all indicators (all *P*adj< 0.001, Table 3). The gain scores, *t* values, Padj values, and effect sizes (Hedges’s g, 95% CI) were as follows: theoretical knowledge test (5.54 ± 2.54 vs. 3.33 ± 1.80, *t* = 6.714, Padj < 0.001, 1.00 [0.61–1.34]), clinical skill operation (6.19 ± 2.01 vs. 3.05 ± 1.66, *t* = 11.127, Padj < 0.001, 1.87 [1.43–2.30]), medical record writing standardisation (5.81 ± 3.32 vs. 2.35 ± 2.55, *t* = 7.246, Padj < 0.001, 1.16 [0.77–1.56]), clinical thinking ability (5.81 ± 3.32 vs. 2.11 ± 2.65, t = 9.063, Padj < 0.001, 1.21 [0.81–1.60]), and doctor-patient communication skills (5.70 ± 2.74 vs. 2.47 ± 2.31, *t* = 7.743, Padj < 0.001, 1.27 [0.87–1.67]). All Hedges’s g values indicated large effects.

**Table 3:** Comparison of gain scores in teaching effectiveness between the two groups.

Dimensions	Group	Gain score (Mean ± SD)	*t* value	*p* value	Hedges’ g (95% CI)
Theoretical knowledge test score	Experimental Control	5.54 ± 2.54 3.33 ± 1.80	6.714	<0.001	1.00 (0.61–1.34)
Clinical skill operation	Experimental Control	6.19 ± 2.01 3.05 ± 1.66	11.127	<0.001	1.87 (1.43–2.30)
Medical record writing standardisation	Experimental Control	5.81 ± 3.32 2.35 ± 2.55	7.246	<0.001	1.16 (0.77–1.56)
Clinical thinking ability	Experimental Control	5.81 ± 3.32 2.11 ± 2.65	9.063	<0.001	1.21 (0.81–1.60)
Doctor-patient communication skills	Experimental Control	5.70 ± 2.74 2.47 ± 2.31	7.743	<0.001	1.27 (0.87–1.67)

### 3.3 Hierarchical linear regression analysis of the effect of the GAiSOLEss teaching model on teaching effect scores

Hierarchical linear regression was used to assess the predictive value of the GAiSOLEs intervention (1 = Control, 2 = GAiSOLEs) for five core training outcomes: theoretical examination scores (TES), clinical skill operation performance (CSOP), standardized medical record writing (SMRW), clinical thinking ability (CTA), and doctor-patient communication competence (DPCC). A two-step hierarchical model was fitted for each outcome independently (consistent criteria across models): Model 1 included control variables (gender, educational background, professional medical background, pre-test scores); Model2 added the intervention group to test incremental predictive validity.

For TES, Model 1 yielded an *R*^2^ of 0.055 and an *F*-value of 1.252 with *p* > 0.05, indicating no significant predictive effect of control variables, while Model 2 achieved statistical significance at *p* < 0.05 with an *R*^2^ of 0.348 and a ΔR^2^of 0.294, and the GAiSOLEs intervention was a significant positive predictor with a *β* of 2.329, a t-value of 6.944 and *p* < 0.001. For CSOP, Model 1 had an *R*^2^ of 0.038 and an *F*-value of 1.069 with *p* > 0.05, showing no predictive power of control variables, and Model 2 reached statistical significance at *p* < 0.05 with an *R*^2^ of 0.562 and a Δ*R*^2^ of 0.524 which represented the largest incremental effect across all outcomes, with the GAiSOLEs intervention acting as a significant positive predictor with a *β* of 2.941, a t-value of 11.363 and *p* < 0.001. For SMRW, Model 1 presented an *R*^2^ of 0.049 and an *F*-value of 1.104 with *p* > 0.05 reflecting a non-significant baseline, Model 2 attained statistical significance at *p* < 0.05 with an *R*^2^ of 0.380 and a Δ*R*^2^of 0.331, and the GAiSOLEs intervention was a significant positive predictor with a *β* of 3.366, a *t*-value of 7.562 and *p* < 0.001. For CTA, Model 1 had an *R*^2^ of 0.066 and an F-value of 1.537 with *p* > 0.05 corresponding to a non-significant baseline, Model 2 reached statistical significance at *p* < 0.05 with an *R*^2^of 0.479 and a Δ*R*^2^of 0.412, and the GAiSOLEs intervention served as a significant positive predictor with a *β* of 3.391, a *t*-value of 9.195 and *p* < 0.001. For DPCC, Model 1 had the lowest baseline variance with an *R*^2^ of 0.036 and an *F*-value of 0.818 at *p* > 0.05 indicating non-significance, Model 2 restored statistical significance at *p* < 0.05 with an *R*^2^ of 0.392 and a Δ*R*^2^of 0.355, and the GAiSOLEs intervention was a significant positive predictor with a *β* of 3.157, a *t*-value of 7.905 and *p* < 0.001 (Table 4).

**Table 4:** Hierarchical multiple linear regression results for primary outcomes.

Indicators	Theoretical knowledge test score		Clinical skill operation		Medical record writing standardisation		Clinical thinking ability		Doctor-patient communication skills	
	Model 1	Model 2	Model 1	Model 2	Model 1	Model 2	Model 1	Model 2	Model 1	Model 2
*R*^2^	0.055	0.348	0.038	0.562	0.049	0.380	0.066	0.479	0.036	0.392
*F* value	1.252	9.535	1.069	27.683	1.104	10.929	1.537	16.363	0.818	11.486
*p* value	0.290	<0.001	0.376	<0.001	0.363	<0.001	0.184	<0.001	0.539	<0.001
Δ*R*^2^		0.294		0.524		0.331		0.412		0.355
Δ*F* value		48.217		129.116		57.180		84.547		62.495
*p* value		<0.001		<0.001		<0.001		<0.001		<0.001

### 3.4 Comparison of digital literacy scores of trainees in the two groups

With regard to trainees’ digital literacy, the experimental group achieved significantly higher scores than the control group across all assessed dimensions, and all intergroup differences were statistically significant (Table 5). To elaborate, the specific scores, t values, Padj values, and effect sizes (Hedges’ g, 95% CI) for each dimension were as follows: digital awareness (experimental group: 18.79 ± 1.50 vs. control group: 17.98 ± 0.88, *t* = 3.513, Padj = 0.001, Hedges’ g = 0.65, 95% CI: 0.28–1.03), digital technology knowledge and skills (11.91 ± 1.81 vs. 10.33 ± 2.36, *t* = 4.017, Padj < 0.001, Hedges’ g = 0.75, 95% CI: 0.37–1.12), digital application (44.88 ± 2.32 vs. 42.58 ± 1.13, *t* = 6.714, Padj < 0.001, Hedges’ g = 1.25, 95% CI: 0.85–1.65), digital social responsibility (25.28 ± 0.92 vs. 24.49 ± 1.75, t = 3.009, Padj = 0.03, Hedges’ g = 0.56, 95% CI: 0.19–0.93), and professional development (16.30 ± 1.25 vs. 15.21 ± 0.84, *t* = 5.444, Padj < 0.001, Hedges’ g = 1.02, 95% CI: 0.63–1.40). Consistently, the total digital literacy score of the experimental group was also significantly higher than that of the control group (117.16 ± 5.08 vs. 110.60 ± 3.37, *t* = 8.126, Padj < 0.001, Hedges’ g = 1.51, 95% CI: 1.10–1.93), further confirming the superior digital literacy of trainees in the experimental group.

**Table 5:** Comparison of digital literacy scores between the two groups.

Dimensions	Experimental group	Control group	*t* value	*p* value	Hedges’ g (95% CI)
Digital awareness	18.79 ± 1.50	17.98 ± 0.88	3.513	0.001	0.65 (0.28–1.03)
Digital technology knowledge and skills	11.91 ± 1.81	10.33 ± 2.36	4.017	<0.001	0.75 (0.37–1.12)
Digital application	44.88 ± 2.32	42.58 ± 1.13	6.714	<0.001	1.25 (0.85–1.65)
Digital social responsibility	25.28 ± 0.92	24.49 ± 1.75	3.009	0.03	0.56 (0.19–0.93)
Professional development	16.30 ± 1.25	15.21 ± 0.84	5.444	<0.001	1.02 (0.63–1.40)
Digital literacy	117.16 ± 5.08	110.60 ± 3.37	8.126	<0.001	1.51 (1.10–1.93)

### 3.5 Comparison of teaching satisfaction scores of trainees in the two groups

The scores of trainees in the experimental group on various dimension indicators such as self-capability improvement, teaching model evaluation and teacher satisfaction were significantly higher than those in the control group, with statistically significant differences (all *p* < 0.001), Table 6.

**Table 6:** Comparison of teaching satisfaction scores between the two groups.

Dimensions	Experimental group	Control group	*t* value	*p* value	Hedges’ g (95% CI)
Self-capability improvement	20.26 ± 4.72	15.88 ± 3.43	5.680	<0.001	1.48 (0.90–2.06)
Teaching model evaluation	29.05 ± 6.87	19.32 ± 3.62	9.466	<0.001	2.47 (1.79–3.16)
Teacher satisfaction	12.74 ± 3.42	9.40 ± 3.62	5.050	<0.001	1.32 (0.75–1.89)

## 4 Discussion

Against the background of digital transformation in health professions education, this study used a randomized controlled design to develop and evaluate the GAiSOLEss instructional model in standardized residency training. After adjusting for baseline equivalence, results showed that GAiSOLEss significantly improved overall teaching effectiveness, residents’ digital literacy, and educational satisfaction compared with conventional training, with the most pronounced improvement in clinical skill operation. Hierarchical multiple linear regression analysis confirmed that these benefits remained statistically significant and independent after adjustment for gender, educational background, and baseline performance, supporting the robust and reliable value of the GAiSOLEss model (14).

A possible explanation for these positive outcomes lies in the standardized closed-loop structure of the six-step GAiSOLEss framework, which integrates knowledge input, practical application, and reflective learning (5, 8, 14, 15). Supported by the DeepSeek-V3-0324 generative AI model, the intervention provides real-time personalized feedback to address delays and inconsistencies in traditional teaching, delivers targeted clinical resources to facilitate systematic knowledge construction, and employs hallucination avoidance procedures to ensure content accuracy (16). In clinical modules such as acute appendicitis training, AI-generated graduated cases combined with group verification activities further strengthen residents’ clinical reasoning ability (17, 18).

For secondary outcomes, residents in the GAiSOLEss group demonstrated superior performance across all subdimensions of digital literacy, particularly in digital application. This trend may be associated with the integrated implementation of GAI throughout the learning process, which could foster digital and data-processing competencies in authentic clinical settings (19). The GAiSOLEss group also reported significantly higher educational satisfaction, reflecting the model’s interactive and personalized design (20).

These favorable results may be linked to the synergistic integration of constructivist learning principles and generative AI technology. By establishing a learner-centered environment that supports self-organized group work, individualized learning plans, and collaborative inquiry (21), GAiSOLEss reduces reliance on passive instruction and promotes self-directed learning and clinical problem-solving. Such features align well with the core goals of contemporary competency-based medical education.

Consistent with previous literature, SOLEs may correlate with enhanced self-directed learning and academic performance, while GAI tools may contribute to improved theoretical knowledge, clinical skills, and case analysis (22). The current study extends this evidence base by integrating SOLEs and GAI into a unified GAiSOLEss model, suggesting that a blended approach yields more comprehensive educational benefits than single instructional methods, especially for clinical skill operation.

Notably, most prior studies on residency digital literacy have relied on standalone courses rather than embedding training within clinical education (22). In contrast, GAiSOLEss integrates digital literacy development into routine clinical skill training, which better matches the demands of modern digitalized clinical practice and offers a feasible strategy to enhance residents’ digital competence. Furthermore, the interactive and personalized design of GAiSOLEss is associated with higher learning satisfaction and engagement, potentially alleviating the rigidity and low motivation commonly observed in traditional residency training.

## 5 Limitations

Several limitations should be acknowledged when interpreting the present findings. First the absence of pre-intervention digital literacy scores and detailed process data on actual AI tool utilization (including usage frequency, duration, task type, and completion rate of monthly themes) precluded adjustment for the potential confounding effects of baseline characteristics and AI tool usage on digital literacy outcomes. Second, lack of process indicators and follow-up was restricted to the training period, and long-term effects on clinical competence and digital literacy were not assessed; thus, an extended follow-up period is needed to examine the sustained impact of the model. Third, the model was developed using a specific AI platform, and its performance may vary when applied with alternative AI systems, suggesting that future studies could compare the effectiveness of different tools within the same framework. Finally, subgroup analyses were not performed in the present study, and further research is required to identify the population that may benefit most from this intervention. Nevertheless, these limitations do not detract from the main conclusions of this study.

## 6 Conclusion

This study provides empirical evidence supporting the potential value of integrating generative AI and self-organized learning environments in standardized residency training. The GAiSOLEss model appears to offer favorable outcomes related to teaching effectiveness, clinical skills, digital literacy, and learning satisfaction, and may represent a feasible approach to inform the digital and intelligent transformation of medical education.

## Data Availability

The raw data supporting the conclusions of this article will be made available by the authors, without undue reservation.

## Footnotes

1
www.randomization.com
