# Supplementary material for: Application of self-organised learning environments integrated with generative AI in standardised training for residents
Source: Front Med (Lausanne). 2026 Mar 16;13:1752647. doi: 10.3389/fmed.2026.1752647 (PMC13033667; doi:10.3389/fmed.2026.1752647)
Supplement: Supplementary file 1 [file Table_1.docx]

**湖北省住院医师规范化培训结业实践技能考核**

**第3站（临床思维）评分表**

考生编号： 得分：

| **评分项目** | | **分值** | **扣分** |
| --- | --- | --- | --- |
| 初始诊断与鉴别诊断（15分） | 初步临床诊断（急性阑尾炎穿孔） | 5 |  |
|  | 诊断依据（转移性腹痛、麦氏点体征、感染指标） | 6 |  |
|  | 鉴别诊断（写出两项即满分，如输尿管结石、异位妊娠、消化道穿孔） | 4 |  |
| 辅助检查安排  （10分） | 血常规（降钙素原等感染指标） | 2 |  |
|  | CRP | 2 |  |
|  | 腹部超声 | 2 |  |
|  | 尿HCG（育龄女性） | 2 |  |
|  | 立位腹平片（排除穿孔） | 2 |  |
| 结合辅助检查结果后给出的临床诊断（15分） | 正确解读超声（阑尾增粗、周围渗出） | 5 |  |
|  | 结合实验室结果（WBC升高、CRP升高） | 5 |  |
|  | 提出CT的必要性（评估穿孔范围） | 5 |  |
| 影像学解读能力（CT）（15分） | 识别阑尾增粗、管壁不规则 | 5 |  |
|  | 发现游离气体及周围渗出 | 5 |  |
|  | 诊断阑尾穿孔伴局限性腹膜炎 | 5 |  |
| 最终诊断与发病机制（18分） | 最终诊断（坏疽性阑尾炎穿孔） | 8 |  |
|  | 发病机制（粪石阻塞→感染→穿孔） | 10 |  |
| 治疗方案（20分） | 急诊手术方式（腹腔镜/开腹切除+引流） | 8 |  |
|  | 抗生素选择（覆盖革兰阴性菌及厌氧菌） | 6 |  |
|  | 术后管理（监测感染、引流护理） | 6 |  |
| 综合评价  （7 分） | 对此疾病的理论基础 | 3 |  |
|  | 表达是否清晰 | 2 |  |
|  | 临床逻辑是否合理 | 2 |  |
| **合计** | | **100** |  |

考官签字： 日期： 年 月 日
