# Supplementary material for: Application of self-organised learning environments integrated with generative AI in standardised training for residents
Source: Front Med (Lausanne). 2026 Mar 16;13:1752647. doi: 10.3389/fmed.2026.1752647 (PMC13033667; doi:10.3389/fmed.2026.1752647)
Supplement: Supplementary file 2 [file Table_2.docx]

**病历书写标准评分表（急性阑尾炎穿孔）**

考生编号： 得分：

| **评分项目** | **具体书写规范及要求** | **分值** | **扣分** |
| --- | --- | --- | --- |
| 一般项目与主诉（10分） | 一般项目（姓名、性别、年龄、住院号等）填写完整、准确，无任何遗漏及错误 | 3 |  |
|  | 主诉表述简洁规范（≤20字），精准涵盖核心症状（转移性腹痛）及病程，无冗余信息 | 4 |  |
|  | 主诉与现病史、初步诊断及最终诊断高度契合，无逻辑矛盾 | 3 |  |
| 现病史（20分） | 详细、规范记录腹痛特征：明确起病时间、诱因、部位（含转移性变化过程）、性质、程度、持续时长及缓解/加重因素 | 6 |  |
|  | 完整记录伴随症状（发热、恶心、呕吐等），明确各症状出现时间、性质及演变特点，无关键症状遗漏 | 5 |  |
|  | 规范记录既往诊疗经过（门诊及外院就诊情况、检查结果、治疗方案及疗效），条理清晰、重点突出 | 5 |  |
|  | 现病史书写逻辑严谨、层次清晰，重点突出，与诊断相关的关键信息无缺失 | 4 |  |
| 体格检查（18分） | 生命体征记录完整、准确，重点记录与感染相关的体温及血压等指标 | 4 |  |
|  | 腹部查体记录详细、准确，明确描述麦氏点压痛、反跳痛、肌紧张程度及肠鸣音变化，无阳性体征遗漏 | 8 |  |
|  | 其他系统查体简要规范记录，无明显阳性体征者需明确注明“未见异常”，无关键查体项目遗漏 | 6 |  |
| 辅助检查（12分） | 完整记录本次所有辅助检查（血常规、CRP、腹部超声等），明确标注检查时间、具体结果，数据准确无误 | 5 |  |
|  | 对各项检查结果进行简要专业解读，结合病情分析其临床意义（如白细胞计数升高提示感染） | 4 |  |
|  | 若行CT检查，需详细、规范记录关键影像学表现（如阑尾增粗、腹腔游离气体等） | 3 |  |
| 诊断部分（15分） | 初步诊断、最终诊断（坏疽性阑尾炎穿孔）书写规范，诊断名称符合临床标准，准确无误 | 6 |  |
|  | 诊断依据充分、严谨，紧密结合病史、体格检查及辅助检查结果，条理清晰、逻辑连贯 | 5 |  |
|  | 鉴别诊断规范完整（至少2项，如输尿管结石、异位妊娠等），明确列出各鉴别要点，具备临床针对性 | 4 |  |
| 诊疗计划（15分） | 手术治疗方案明确、规范，明确标注手术方式（腹腔镜/开腹阑尾切除术+腹腔引流术） | 5 |  |
|  | 抗生素使用方案合理，明确标注抗生素种类（需覆盖革兰阴性菌及厌氧菌）、给药途径及使用时长 | 5 |  |
|  | 术后管理计划具体、规范，明确包含感染监测、引流管护理、对症支持治疗等关键措施，无遗漏 | 5 |  |
| 书写规范（10分） | 字迹清晰、工整，无涂改、刮擦痕迹；医学术语规范准确，无错别字及语法语病 | 5 |  |
|  | 全文逻辑严谨、层次分明，严格遵循病历书写相关规范，无重要临床信息遗漏 | 5 |  |
| **合计** | | **100** |  |

考官签字： 日期： 年 月 日
